# Advice from a systems-biology model of the corona epidemics

**DOI:** 10.1038/s41540-020-0138-8

**Published:** 2020-06-12

**Authors:** Hans V. Westerhoff, Alexey N. Kolodkin

**Affiliations:** 1 Infrastructure for Systems Biology Europe - The Netherlands (ISBE.NL), Amsterdam, The Netherlands; 2 Molecular Cell Biology, VU University Amsterdam, Amsterdam, The Netherlands; 3 Synthetic Systems Biology and Nuclear Organization, Swammerdam Institute for Life Sciences, University of Amsterdam, Amsterdam, The Netherlands; 4 Manchester Centre for Integrative Systems Biology, Manchester, UK; 5 Luxembourg Centre for Systems Biology, Luxembourg City, Luxembourg

**Keywords:** Immunology, Computer modelling, Cancer, Decision making

## Abstract

Using standard systems biology methodologies a 14-compartment dynamic model was developed for the Corona virus epidemic. The model predicts that: (i) it will be impossible to limit lockdown intensity such that sufficient herd immunity develops for this epidemic to die down, (ii) the death toll from the SARS-CoV-2 virus decreases very strongly with increasing intensity of the lockdown, but (iii) the duration of the epidemic increases at first with that intensity and then decreases again, such that (iv) it may be best to begin with selecting a lockdown intensity beyond the intensity that leads to the maximum duration, (v) an intermittent lockdown strategy should also work and might be more acceptable socially and economically, (vi) an initially intensive but adaptive lockdown strategy should be most efficient, both in terms of its low number of casualties and shorter duration, (vii) such an adaptive lockdown strategy offers the advantage of being robust to unexpected imports of the virus, e.g. due to international travel, (viii) the eradication strategy may still be superior as it leads to even fewer deaths and a shorter period of economic downturn, but should have the adaptive strategy as backup in case of unexpected infection imports, (ix) earlier detection of infections is the most effective way in which the epidemic can be controlled, whilst waiting for vaccines.

## Introduction

Different governments take different measures vis-à-vis the COVID-19 crisis, ranging from advice to reduce social activities, to a complete lockdown of society and economy. Many governments do not seem to benefit maximally from the experiences of other countries. Almost invariably measures are taken too late. In this epidemic, times are too short for maximally informed, well-balanced deliberations leading to optimal and early conclusions. Policymakers, members of parliament, and voters, all require tools that enable them to anticipate better and to then fulfill their tasks. We here provide such a tool and we show how this tool leads to conclusions that could well prove crucial for managing the epidemic. We also delineate a new adaptive control method.

## Results

**Fig. 1:**
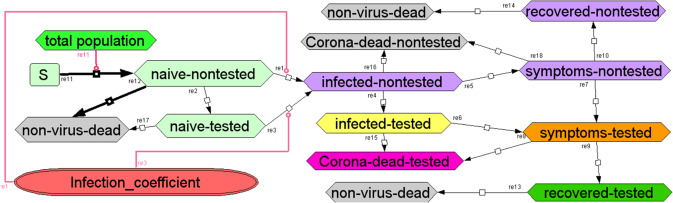
Systems biology model of the Corona virus epidemics. Species are in boxes, reactions (irreversible, mass-action) are indicated by arrows with reaction numbers written alongside. The infection coefficient is a linear function of the numbers of infected-nontested, infected-tested, symptoms-nontested and symptoms-tested, with relative weights 0.508, 0.25, 0.025 and 0.025 respectively divided by a fitted number close to the total population number of the geographical unit, and a social distancing factor of 1 (in case of no lockdown), 10 (used to describe ‘total’ lockdown) or anywhere in between. Model with all parameters values is at 10.15490/fairdomhub.1.model.693.1.

Using standard systems biology methodology we generated a dynamic model, which should apply to various geographical units after adjustment to the population size. We use the term ‘geographical unit’ for an area where the lockdown intensity is fairly homogeneous. This may be a country, a state, or even a city or village. For each of the processes depicted in Fig. 1, the model uses a chemical reaction equation (describing the conversion that occurs in the process) and a rate equation (describing the rate at which this occurs in terms of a rate ‘constant’ [probability] and concentrations of species (such as infected-non-tested individuals)).

**Fig. 2:**
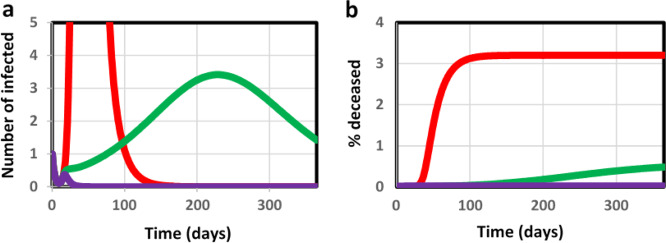
Predicted progression of the epidemic. Predicted progression of epidemic during the first year in terms of **a** number of infected that test positively and **b** the % deceased individuals due to the virus; without government action (red), with social distancing at factor 2.2 (green) and with complete lockdown (i.e., social distancing factor of 10; purple). Lockdown measures were taken at *t*=15 days; hence the curves coincide before this time.

**Fig. 3:**
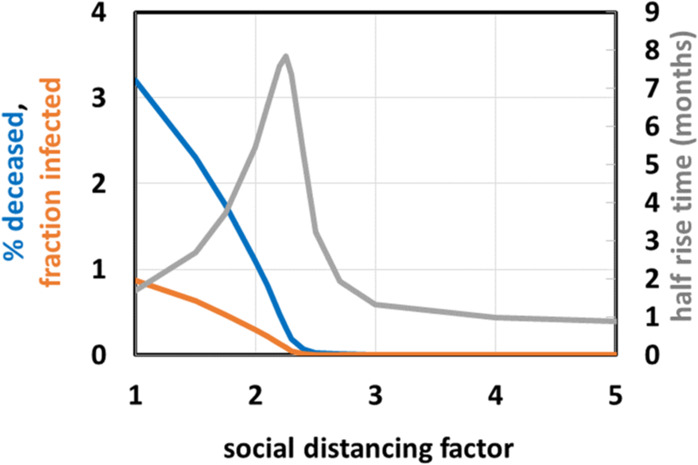
Effect of variation of the lockdown intensity. Effect of lockdown as function of lockdown intensity (expressed in terms of social distancing factor): % deceased (blue), fraction (i.e., %/100) infected (orange) both calculated at the end of the epidemic, and half rise time of the epidemic (in months; gray).

For the unmanaged epidemic, 3% of the population is computed to die from COVID-19 infection within 3 months (red line in Fig. 2b). A ‘complete shutdown’ at *t* = 15 days into the epidemic (modeled as a permanent reduction of infection probability by a factor of 10 through a ‘social distancing factor’ [see below] of 10) should keep the percentage of deceased individuals down to 0.003% (400 times lower than the natural death rate per year; purple line in Fig. 2b). In our model simulations, a less complete lockdown increased lethality in a highly nonlinear way (Fig. 3). Dividing the fraction of the population that becomes infected, by the fraction deceased, both as shown in Fig. 3, we obtained a proportionality constant independent of the intensity of the lockdown, and equal to 27 (which reflects the inverse of the lethality of the disease). Consequently, in order to achieve the 50% herd immunity that should quench this and a subsequent wave of the epidemic, one would have to accept a death rate of 2% over some 5 months. This corresponds to approximateley three times the natural death rate: adjusting the lockdown intensity so as to obtain sufficient herd immunity may thereby be unacceptable ethically.

Acceptable strategies should perhaps focus on measures that keep the death toll around 0.03%/year, i.e., well below the natural death rate. Because the terminally ill occupy an intensive care bed for approximately a month with half of them surviving, and assuming a peak of the epidemic lasting 3 months (see below), this corresponds to approximately 2 such beds for every 10 000 inhabitants, close to the actual total intensive care capacities in Northern Europe. The blue line in Fig. 3 shows the computed COVID-19 mortality as a function of the intensity of the lockdown. The lockdown intensity is parameterized by the ‘social distancing factor’ which we define as the factor decrease in infection coefficient (Fig. 1) brought about by the lockdown measure called by the government of the geographical unit. A 2.5 fold permanent reduction in social interactions (i.e., a social distancing factor of 2.5) should reduce COVID-19 mortality from 3% to this 0.03% (the blue line in Fig. 3). However, the duration of the epidemic (measured as the half rise time) varies appreciably with the social distance. Around a 2.2 fold increase in social distance the duration of the epidemic is predicted to be the longest (more than half a year, see the gray line in Fig. 3 at social distancing factor of 2.2). This has the benefit of minimizing the challenge on the intensive care unit capacity, but may well increase the economic damage.

A full lockdown should reduce the duration of the epidemic to 25 days (0.8 months, see gray line in Fig. 3), with a stronger but short effect on the economy (but see below for an extra strategy that will be needed to prevent re-emergence of the epidemic). On the other hand, a 3-fold decrease in social contact (i.e., implementation of a lockdown with an intensity of a social distancing factor of 3) should already reduce the duration to 40 days (1.3 months, gray line in Fig. 3), with as additional benefit a much reduced lethality (0.008% compared with 0.5 at a 2.2 fold reduction of infection probability; blue line in Fig. 3) and much reduced economic damage when compared to the situation of a social distancing factor of 2.2). From this we conclude that a soft lockdown such as corresponding to a 2.2 fold reduction in social interaction could be the worst strategy to follow, that a stronger lockdown is advisable and that that lock down need not be that much stronger (e.g. a social distancing factor of 3 rather than 2.2 should do; see Fig. 3). It should be noted that in all these cases the herd immunity reached within a year will not suffice to prevent a second wave of epidemic.

**Fig. 4:**
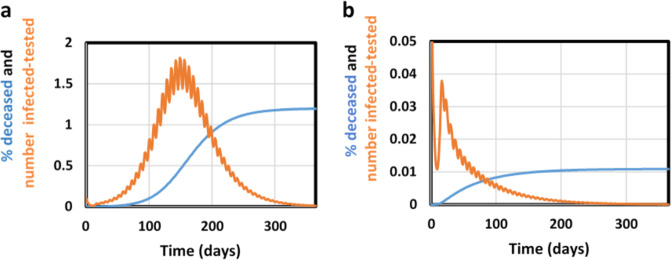
Intermittent lockdown scenarios; % deceased (blue) and number of infected-tested/10 (orange) as functions of time during the first year. **a** Intermittent lockdown 55% down/45% up and **b** Intermittent lockdown 70% down/30% up.

A strong lockdown is hardship. Therefore we examined whether such a lockdown could be intermitted with periods with normal social contact, without endangering the success of the strategy. We found (Fig. 4a) that a 55%-on-45%-off schedule for the full lockdown will not suppress the epidemic. In order to suppress the SARS-CoV-2 virus, two thirds of the time society should be locked down, leaving one third for social interactions (Fig. 4b and supplementary material Fig. S1). This seems an attractive alternative to a permanent lockdown provided a selection of economic activities that require live human-human interactions could be confined to shorter time periods without increasing contact intensity.

**Fig. 5:**
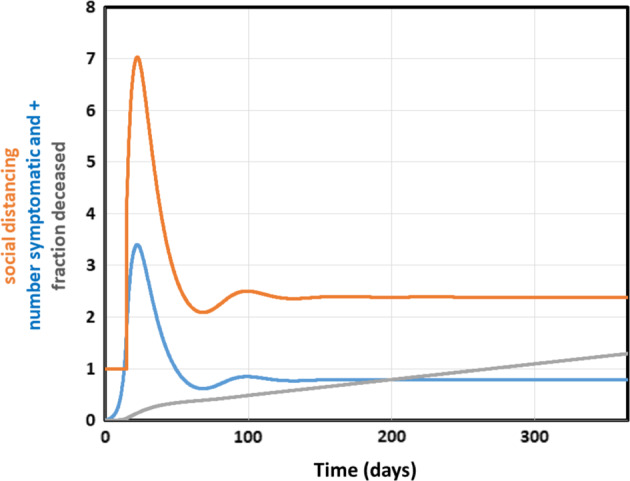
Adaptive lockdown instated at t=15 days, followed for a year. Number of symptomatic individuals tested positive (=controlling variable, /100, blue), social distancing factor (orange) and % deceased (*100, gray line). After *t* = 15 days, a lockdown of varying strength was instated, by making the social distancing factor a function of the number of people with syptoms newly tested positive for Corona, according to 1 + 0.0177*symptomatically-tested.

A more complex strategy should be one where the intensity of the shutdown is adapted on the fly to the severity of the epidemic. Choosing the fraction of the population that is newly tested as virus-positive (blue line in Fig. 5) as the variable controlling the social distancing factor as shown by the orange line in and legend to Fig. 5, this adaptive strategy should do better than a fixed lockdown of comparable intensity. Implemented at time 15 days after the first detection of an infected individual, this should lead to a lethality after one year of only 0.013% (gray line in Fig. 5), i.e., one fourth the 0.33% a continuous 2.25 fold lockdown would have led to (see Fig. 3). A disadvantage of this adaptive strategy is that the mortality increases linearly with time also after the first year. However, the total mortality should still not overtake that of the constant lockdown by social distancing factor of 2.25 until after 10 years. We reckon that long before then a vaccine, some other cure, or an improved patient detection and insulation strategy should have been discovered and put in place. The adaptive lockdown could be optimized further in terms of parameters and with respect to any specific epidemic, culture and geographical unit.

The blue line in Fig. 5 shows that for this adaptive strategy the effect on the number of individuals tested positively should be noticeable immediately after its onset: initially at least, the strategy comes with a rather intensive lockdown, much stronger than what many governments are practicing judging by the slower rate of decrease in percentage infected reported for Europe and North America^1^. Most governments do the inverse: they exercise a soft lockdown first, increasing the lockdown intensity subsequently (see also below). The adaptive lockdown strategy proposed here begins with a harsh lockdown to then relax it. The latter is the one that should work.

One of the important determinants of the success of a lockdown is how early in the epidemic it is enforced. This is even so for the adaptive lockdown strategy. Should the strategy be enacted 15 days later than modeled here, the number of dead at the end of the year would become 20 times higher and the required initial lockdown intensity should become 500 (in terms of social distancing factor), even though ultimately the adapting lockdown level should subside to the same social distancing factor of 2.2. So, governments should act earlier rather than later.

**Fig. 6:**
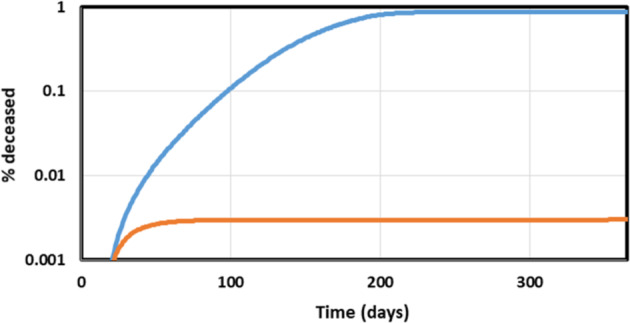
The effects of the sequence of lockdown on the fraction of the population dying from the disease as a function of time. At time 15 days a lockdown was instated either of social distancing factor 2 (‘soft’) or of social distancing factor 10 (‘strong’). At time 190 days the lockdown intensity was altered to a social distancing factor of 10 and 2, respectively. The ‘soft-then-strong’ lockdown strategy is shown by the blue line, the ‘strong-then-soft’ strategy by the orange line.

This phenomenon is reinforced by comparing a ‘soft-then-strong’ lockdown strategy that starts with a mild lockdown and is then followed by a harsh lockdown, to an inverse strategy, i.e., first harsh and then mild. Figure 6 shows that the effects of the two strategies differ immensely: for social distancing factors of 2 and 10 for instance, the harsh-then-soft lockdown leads to a lethality of 0.003% whereas 0.9% of the population would die from the corresponding soft-then-harsh strategy.

Faster testing of the *symptomatic* individuals should have little effect (results not shown), but a faster detection of symptoms in the individuals that have been infected but are not yet symptomatic should be highly effective: Doubling this rate constant reduces lethality after one year from 0.013% to 0.0030% and reduces the required peak in the adapting social distancing factor to only 1.4, thereby strongly reducing the economic damage. Quadrupling the same rate constant reduces the distancing to a factor smaller than 1.1 and the % deceased after a year to 0.00012%. This same method should help detecting people that import the disease virus from abroad. Quarantine of the newly arrived persons should be effective, but should the entry of the infected people not be noticed, then the adaptive strategy should take care of it provided there is testing (results not shown): in the adaptive strategy the number of infected individuals is closely related to the control variable of the adaptive system.

An attractive alternative to the adaptive strategy is the full lockdown until virus extinction. Here the lockdown strategy must be intensive and continued until there are no infected people left in the population (results not shown). However, this strategy is sensitive to import of new infections (Supplementary Fig. S2) and should only be robust if an adaptive strategy serves as back up.

The adaptive strategy does have the disadvantage that it should be maintained until a vaccine, or a much faster detection method of the infections, or another effective way of reducing the infection coefficient, has arrived. Stopping the adaptive control at 180 days after onset, had the effect that the epidemic re-emerged with a half rise at 230 days, i.e., only one and a half month later (Supplementary Fig. S3). The adaptive control method is ill-compatible with the extinction strategy however, as in the latter there is no *live* control variable left. Yet the adaptive strategy can be used as back up, provided a suitable and fast control variable is identified.

## Discussion

Simulations using a quantitative model of the COVID-19 epidemic as a tool herewith showed that strategies aiming for herd immunity are unacceptable and that initially a much stronger lockdown is required than what is practiced by many governments. The argument that this strategy does not protect against imported virus infections, is irrelevant for *this* epidemic, as herd immunity will develop too slowly and at too high a death toll. Similar conclusions have also been achieved by more complex models (e.g., 7). The advantage of the present model is that it is public domain, can be used by anyone with a personal computer, and the parameters that it uses are accessible. By surprising contrast, models used by some government advisers, are kept secret; model assumptions, rate equations and parameter values are thereby not accessible to scientific assessment. This paper further developed an adaptive strategy that should be superior in terms of epidemiology over the fixed lockdown strategy and that deals with infections from abroad.

Our results suggest that the measures taken by many policy makers will be insufficient to quench the epidemic at acceptable death tolls. Some Western policy makers engage in an adaptive lockdown strategy but one of insufficient strength, or in the absence of a control variable other than public pressure towards lockdown mitigation: our results are further consistent with the observation that the intial tendency towards a slowly increasing lockdown strategy is ill-effective (Fig. 6). What is necessary is a harsh lockdown at first, which may then be softened as the number of infected individuals begins to decrease with time but only on the basis of measuring this (Fig. 5). Policy makers in Taiwan and South Korea have been able to quench the epidemic in this or harsher ways. The argument expressed by some European policy makers that such measures cannot be taken in democracies is thereby nullified. The Chinese measures appear to be closest to the full lockdown and virus-eradication strategy and have been most successful, but may also have been harsh on society. And certainly now that the remaining worry is import of new virus, China may be best off with quarantine of all visitors from abroad, with the adaptive strategy modeled here as a backup.

The adaptive and the harsh-then-soft strategies have the advantage that the general public once confronted with the first signs of a terrible epidemic may accept a harsh lockdown at first and then be assuaged when the lockdown is softened gradually. If the public first meets a soft lockdown, which then needs to become more and more intensive, it may readily become impatient and disappointed in not being rewarded for its engagement with the lockdown, by a subsequent softening thereof. The intermittent strategy may be confusing to the general public at first, yet become readily acceptable because people can still do their most urgent things be it in limited periods of time. People may readily associate this to the week-weekend time schedule they are anyway used to.

Our model comes with a number of limitations. It assumes homogeneity within the modeled population, e.g. that in all regions within the modeled geographical unit any lockdown is equally effective, that all fractions of the population are subject to the same infection parameters, that all days of the week are the same, that there are no policy and therapy changes in time, that the lethality of the virus does not change from one age group to another. None of these assumptions are correct, but the correspondence between our results and observations worldwide may suggest that the limitations may not matter much. On the other hand, the epidemics in some countries seems to persist longer than perhaps expected on the basis of the intensity of the lockdown. This may be because pockets in society are not fully engaged in the lockdown. In some countries for instance, health care workers have continued to care for the elderly without proper protective measures such as mouth caps, simply for the lack of the latter or for reasons of faltering directives. It should be easy technically to make new versions of the present model that take these heterogeneities into account; the limiting steps here reside more in obtaining the appropriate parameter values. Therefore we think that this needs to be postponed to separate publications. Then there is the limitation that our model uses a continuous description in terms of real numbers and probabilities, whereas numbers in the true epidemic can drop below 1, making infection probabilities not just small but zero. We have developed a version of the model that contains this property and used this to examine the effect of imported COVID-19 cases (Supplementary Fig. S2). As expected the infection essentially returned the model virtually to the continuum case. Since the great majority of geographical units are far from such an eradication of the COVID-19 epidemic, we have not here elaborated upon this more stochastic version of the model.

Since the submission and reviewing of this paper, the epidemics has reached a peak in many countries and some countries are relaxing their lockdown measures. Also here our model may be helpful, first because it shows how an adaptive lockdown where the adaptation is determined by a control variable which measures the progress of the epidemic, will work. Additional simulations for when the lockdown-release is non adaptive show the high risk if such a control variable is not engaged in the process. It is a major worry therefore that some governments are easing the lockdown in the absence of effective and fast measurements of how the epidemic is progressing. Second, our model may be used to compare the effectiveness of different lockdown-release strategies in different geographical units.

## Methods

The construction of the diagram for the model of the COVID-9 epidemics used the open access CellDesigner software (v4.4; Systems Biology Institute, http://celldesigner.org/index.html)^2^. The network diagram was translated into a dynamic model using the open access software called COPASI (www.copasi.org)^3^. The resulting model was stored in the model/data repository FAIRDOMHub (10.15490/fairdomhub.1.model.693.1)^4^, within the FAIRDOMHub investigation 10.15490/FAIRDOMHUB.1.INVESTIGATION.372.1^5^ Model parameters are in this model as deposited. They and their sources are specified in the supplementary material. In addition, the model is available for direct use at JWS Online, where parameter values are completely open to be used, manipulated, and improved by others (https://jjj.bio.vu.nl/models/westerhoff1/simulate/)^6,7^. Other, more complex models exist and have come with some similar conclusions^8^, do not address all the issues addressed here and do not provide accessibility to systems biology tools by being SBML-convertible and accessible to Copasi and JWS-online. Our model and its conclusions are also presented as YouTube movie^9^.

### Reporting Summary

Further information on research design is available in the Nature Research Reporting Summary linked to this article.

## Supplementary information

Supplementary material

Reporting summary

Dataset 1

Dataset 2

Dataset 3

Dataset 4

Dataset 5

Dataset 6

## Data Availability

The model inclusive of its parameter values is publically available: It has been stored in the model/data repository FAIRDOMHub (10.15490/fairdomhub.1.model.693.1)^4^ within FAIDROMHub investigation (https://fairdomhub.org/investigations/372)^5^. The model is also available for online simulations at JWS Online, where parameter values are completely open to be used and improved by others (https://jjj.bio.vu.nl/models/westerhoff1/simulate/)^6^ and the results are explained through a video presentation (https://youtu.be/lbsqJ1_WvmE)^9^. The particular models used for each figure in the main text are in the GitHub: https://github.com/HansWesterhoff/Coronapaper-March-2020. All data used came from public data sources.
