# Supplementary material for: Advice from a systems-biology model of the corona epidemics
Source: NPJ Syst Biol Appl. 2020 Jun 12;6:18. doi: 10.1038/s41540-020-0138-8 (PMC7293297; doi:10.1038/s41540-020-0138-8)
Supplement: Supplementary file 1 — Supplementary material [file 41540_2020_138_MOESM1_ESM.pdf]

Supplementary material of:

Advice from a systems-biology model of the Corona epidemics

Hans V. Westerhoff[1-4,*] and Alexey N. Kolodkin[2,5]

(**NPJSBA-00562R; supplemental material**)

[1]Infrastructure for Systems Biology Europe (ISBE), EU

[2]Molecular Cell Biology, VU University Amsterdam, THE NETHERLANDS;

[3]Synthetic Systems Biology and Nuclear Organization, Swammerdam Institute for Life Sciences, University of Amsterdam, THE NETHERLANDS

[4]Manchester Centre for Integrative Systems Biology, UK

[5]Luxembourg Centre for Systems Biology, Luxembourg

*Corresponding author at hans.westerhoff@manchester.ac.uk

Supplementary figures 2

COVID-19 model parameters 3

- Parameter values 3
- Sources: 4

Location of the Copasi modelfiles 6

Models and main-text figures reproduction 6

- Figure 1. Model diagram 6
- Figure 2. Epidemic dynamics 7
- Figure 3 Social distancing dependence 8
- Figure 4 Intermittent lockdown 9
- Figure 5. Adaptive lockdown 10
- Figure 6. Strong then soft better than soft then strong 11

Supplementary figures

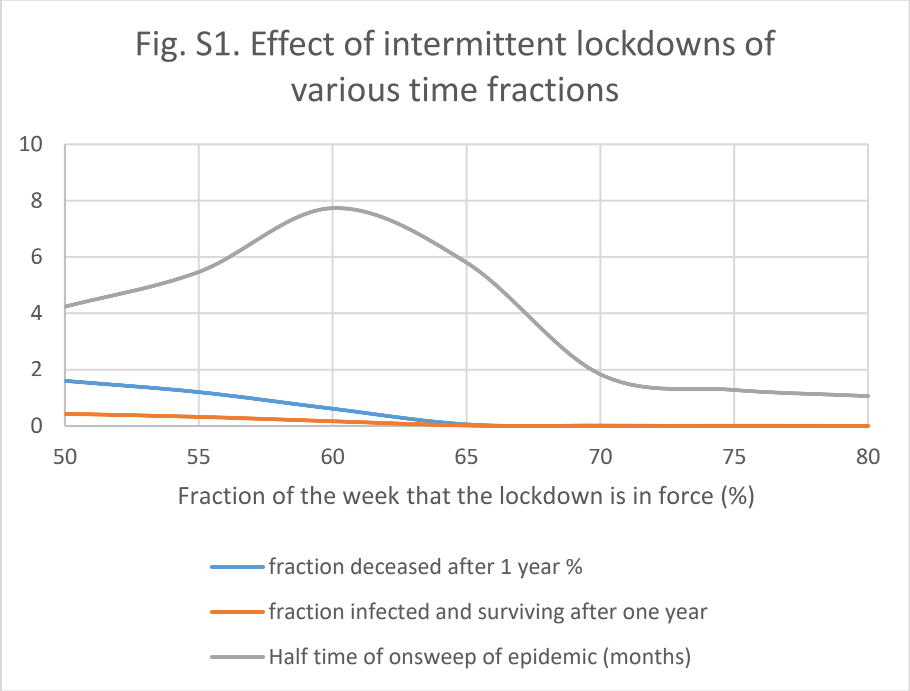

Figure S1. Fraction dead, cured and epidemic rise time as function time fraction of intermittent lockdown.

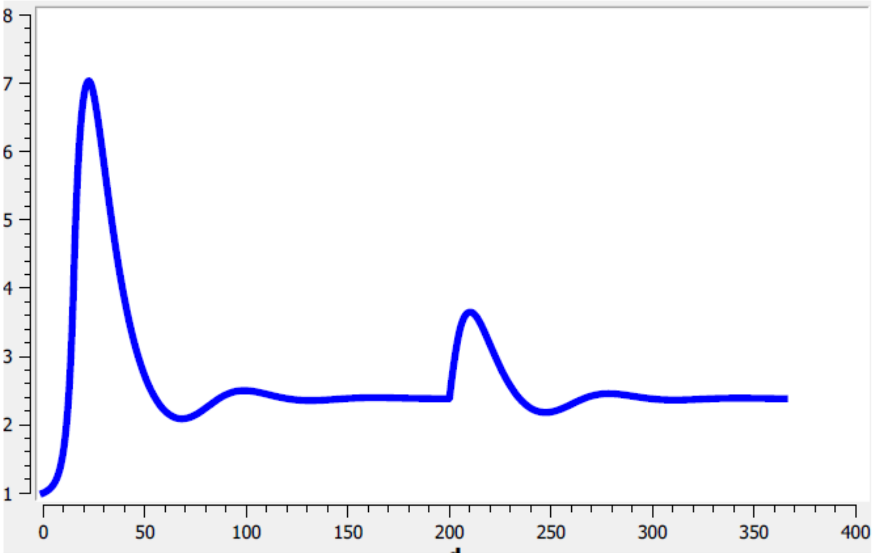

Figure S2. Social distancing factor effected by the adaptive model plotted versus time in days, with a foreign re-infection occurring at t=200 days with 50 new cases.

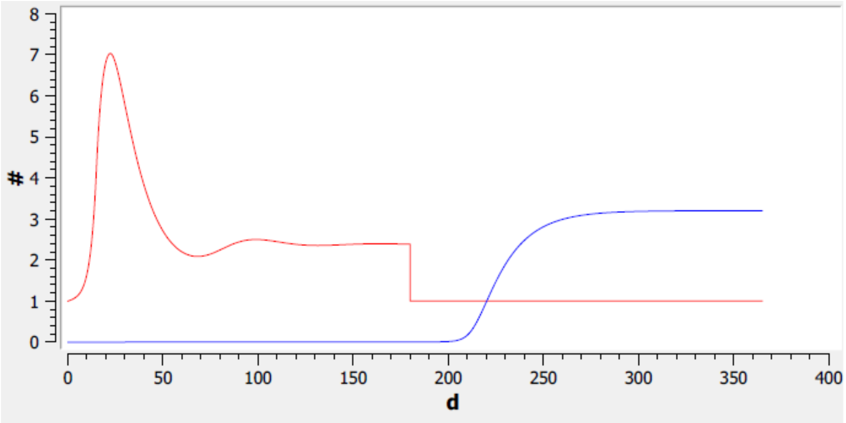

Figure S3. Epidemics when ending adaptive control at t=180. Blue: % deceased. Red: Social distancing factor implemented by the adaptive lockdown algorithm. Both plotted versus time in days.

COVID-19 model parameters

Parameter values

Reaction N	Name	Reaction		in model	Source	units
				S=1 (fixed)		
				T=630,000	source1	persons
11	birth	S -> uninfected_non-tested; "total population"	v=S*T*k	k=0.0000329	Source2	born per person per day
17	Normal Death	uninfected-tested -> non-corona-dead				
14	Normal Death	recovered-nontested -> non-corona-dead				
13	Normal Death	recovered-tested -> non-corona-dead				
12	Normal Death	uninfected_non-tested -> non-corona-dead	v=S*k	k=0.0000329	balanced with re 11	death per person per day
18	COVID death	symptoms-nontested -> Corona-dead-nontested				
16	COVID death	infected-nontested -> Corona-dead-nontested				
15	COVID death	infected-tested -> Corona-dead-tested				
8	COVID Death	symptoms-tested -> Corona-dead-tested	v=S*k	k=0.02	computed, Source3	death per person per day
10	Recover	symptoms-nontested -> recovered-nontested				
9	Recover	symptoms-tested -> recovered-tested	v=S*k	k=0.067	computed Source4	recovered per person per day
1	Infection	uninfected_non-tested -> infected-nontested	v=S*k			

3	Infection	uninfected-tested -> infected-nontested		k=infection coefficient, depends on a social distance factor as described below, on the numbers of the four types of individual infected, and on the four corresponding infection probabilities, , as described in the legend to Fig. 1.		
5	Symptoms	infected-nontested -> symptoms-nontested				
6	Symptoms	infected-tested -> symptoms-tested	v=S*k	k=0.3	Source5	per person per day
4	Testing	infected-nontested -> infected-tested				
2	Testing	uninfected_non-tested -> uninfected-tested	v=S*k	k=0.0008	Source6	per person per day
7	Testing Sympt	symptoms-nontested -> symptoms-tested	v=S*k	k=1	Source7	per person per day

The 'infection coefficient' is defined by the probability that a healthy person meets another person in society (which is essentially taken as the inverse of 'Social_Distance') multiplied by the probability that this encounter leads to infection. It is taken to equal the long formula:

("[infected-nontested]" ·"Values[Infection_from_non-tested_no-symptoms].InitialValue" + "[symptoms-nontested]" ·"Values[Infection_from_non-tested_symptoms].InitialValue" + "[infected-tested]"
Social_Distance

nitialValue" + "[infected-tested]" ·"Values[Infection_from_tested_no-symptoms].InitialValue" + "[symptoms-tested]" ·Values[Infection_from_tested_symptoms].InitialValue) ·(1 - "[epidemic extinguished]")
Social_Distance

The 'Social_Distance' is the number of people in the geographical unit, but slightly adjusted (here from 630 000 to 534 700) to fit the initial exponential increase). At the moment of government interference, the Social Distance is multiplied with the social distancing factor:

$$10 \cdot \left(1 + \frac{\frac{2.5 \cdot 141}{141} \cdot \text{"[symptoms-tested]"}}{\text{"Values[symptomatic number tolerance].InitialValue"}} \cdot \text{"Values[government response factor to diagnosed infected].InitialValue"}\right)$$

Because, in this application of the program the factor on the right-hand side equals zero, this equals 10 in the default version of the program. To adjust the 'social distancing factor' the number 10 is put equal to the actual social distancing factor (typically 1 for no government interference, 2.5 for intermediate lockdown and 10 for 'complete' lockdown).

Sources:

Source 1. Population number
This is close to the city of Rotterdam (EU, 650,000)
https://en.wikipedia.org/wiki/Demography_of_the_Netherlands

Source 2 Rate of birth
Close to the data from the Netherlands and therefore likely for Rotterdam(https://en.wikipedia.org/wiki/Demography_of_the_Netherlands)

169015 [new born]/ 17282000 [Total population]=0.00978 [born per person per year] = 0.0000268 [born per person per day]

Source 3. Rate of death

Death for COVID-19 patients=Normal Rate of death + 0.03/15 = 0.002 (3% chance to die within 15 days). This gives the following number of deaths close to what was actually observed initially in various countries:

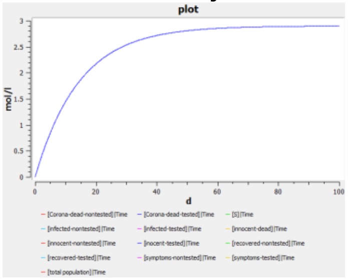

Source 4 Rate of recovery

To obtain recovery time close to what was observed in [https://www.thelancet.com/action/showPdf?pii=S0140-6736%2820%2930566-3, Fig 1] , and balances with COVID death rate.

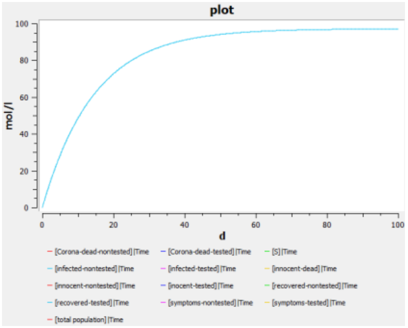

Source 5. Symptoms development

Balanced in such a way that half of infected develop symptoms within 5 days and almost all infected develop symptoms within 10 days (red line), i.e. similar to observed at [https://www.thelancet.com/action/showPdf?pii=S0140-6736%2820%2930566-3, Fig 1]

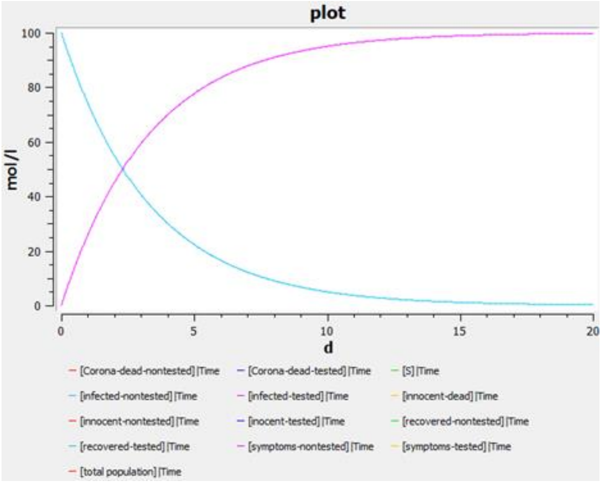

Source 6. Rate of testing for naïve and infected without symptoms
k_testing =0.0008 [per person per day|

Source 7. Rate of testing for infected with symptoms
k_testing = 1, such that almost all will be tested within 2 days

Location of the Copasi modelfiles

The models referred to below can be found as Copasi code on the GitHub
https://github.com/HansWesterhoff/Coronapaper-March-2020

Models and main-text figures reproduction

All figures (except Figure 1 were made in Copasi, then in excel. Al figures were converted to PNG and then (using https://www.online-convert.com/result/7749ebb5-d407-4b62-95e9-6238367aa147) to EPS files.

Figure 1. Model diagram

The construction of the diagram for the model of the COVID-9 epidemics used the open access CellDesigner software (v4.4; Systems Biology Institute, http://celldesigner.org/index.html). The picture was stored as PNG file (Figure 1 PNG), PDF (Figure 1 pdf) and as HTML (Figure 1 HTML) file on the GitHub https://github.com/HansWesterhoff/Coronapaper-March-2020.

The figure 1 is:

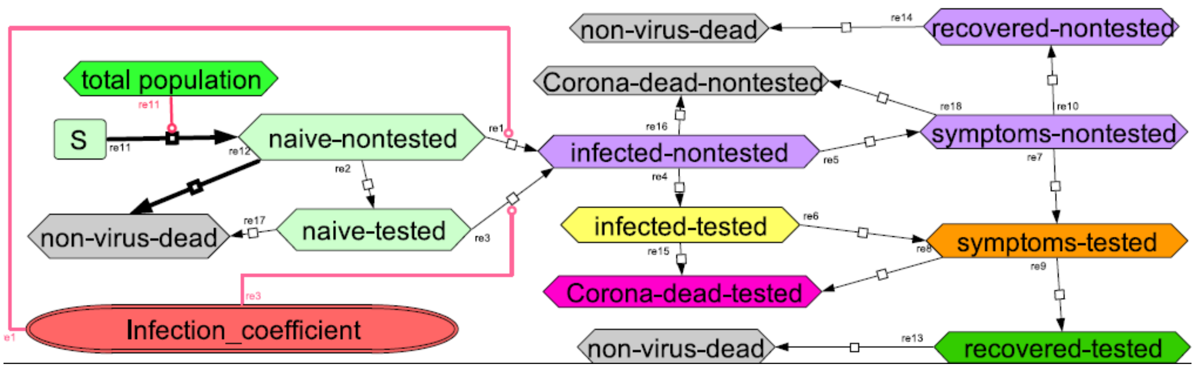

Figure 2. Epidemic dynamics

The blue curves are for a *social distancing factor [here called government response factor to diagnosed infected)* of 10 in the model '*Fig2 blue sdfactor 10 Wff1Corona_Lux_2020-03-20_1340 intermittentGwork full lockdown effect of socialdistancing Fig 3 also Fig 2C.cps*'. saved as 'Figure 2 sdf=10 Copasi'

The two blue/purple figures A and B are then obtained by running the time mode of the Copasi file and looking at the plots produced.

In the analogous manner the green lines in figures 2A and 2B for a soft lockdown of factor 2.2 are produced by changing the factor 10 in the formula (which is the social distancing factor we used for 'no lockdown' as we assume that the occurrence of the epidemic will automatically make people more wary), for the *social distancing_factor* to 2.2. The Copasi file '*Fig2 green sdfactor 10 Wff1Corona_Lux_2020-03-20_1340 intermittentGwork full lockdown effect of socialdistancing Fig 3 also Fig 2B.cps*' (copied to 'Figure 2 sdf=2.2 Copasi' has this done already.

And then for lack of any government interference the factor 10 in the social distancing factor (which in the computer model is called '*Government_induced_isolation_factor')* is set to 1 and the two orange curves are produced. The Copasi file '*Fig2 orange sdfactor 10 Wff1Corona_Lux_2020-03-20_1340 intermittentGwork full lockdown effect of socialdistancing Fig 3 also Fig 2A.cps*' (copied to Figure 2 cdf=1' has this done already.

Excel file is 'Figue 2 Excel'.

Figure 2 was *actually* obtained by using the Copasi files:

Wff1Corona_Lux_2020-03-20_1340 intermittentGwork full lockdown effect of socialdistancing Fig 3 also Fig 2WORK2A copied to 'Figure 2 sdf=1'

Wff1Corona_Lux_2020-03-20_1340 intermittentGwork full lockdown effect of socialdistancing Fig 3 also Fig 2WORK2B copied to 'Figure 2 sdf=2.2'

Wff1Corona_Lux_2020-03-20_1340 intermittentGwork full lockdown effect of socialdistancing Fig 3 also Fig 2WORK2C copied to 'Figure 2 sdf=10'

which are all the same except for the setting of the parameter 'Government_induced_isolation_factor' where the pre-multiplier was set to 1, 2.2 or 10, for the 'without government action', 'factor 2.2 social distancing' and 'complete lockdown', respectively (the 141/141 is a historical artifact in the model):

$$1 \cdot \left(1 + \frac{\frac{2.5 \cdot 141}{141} \cdot \text{"[symptoms-tested]"}}{\text{"Values[symptomatic number tolerance].InitialValue"}} \cdot \text{"Values[government response factor to diagnosed infected].InitialValue"}\right)$$

The Copasifiles all have time steps of 1 day in order to keep the excel file manageable. The files have a complex title because they are capable of many other features that are not utilized here, but will be utilized in other figures of the paper.

Each file calculates for one parameter setting, i.e. either without government action ('WORK2A'), with factor 2.2 social distancing ('WORK2B') or with complete lockdown ('WORK2C'). The text files are output from the computation of the figure '% dead, #infected_tested and #symptoms_not_tested versus time (days)', but then only retaining '% corona dead' (Fig. 2B) and 'infected tested' (Fig. 2A): Confusingly the final figures entitled 2A and 2B each show one aspect for the three cases (e.g., this '2A' does not correspond to the 'New2A'). This is what the excelfile achieves. This excel file has been copied to 'Figure 2 Excel'.

Files are on the GitHub https://github.com/HansWesterhoff/Coronapaper-March-2020

The figure 2 is:

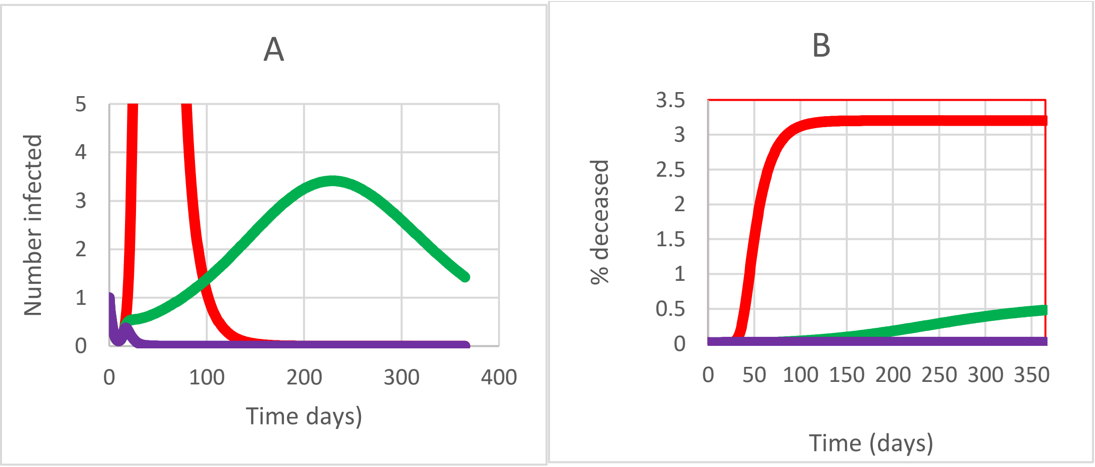

Figure 3 Social distancing dependence

The data were obtained by running the file 'Wff1Corona_Lux_2020-03-20_1340 intermittentGwork full lockdown effect of socialdistancing Fig 3 also Fig 2WORK2A' (copied to Figure 3 Copasi) for

various social distancing factors, again by using the pre-multiplier (1. in the expression below) of the expression for the ‘Government_induced_isolation_factor’:

$$1\cdot\left(1+\frac{\frac{2.5\cdot 141}{141}\cdot\text{"[symptoms-tested]"}}{\text{"Values[symptomatic number tolerance].InitialValue"}}\cdot\text{"Values[government response factor to diagnosed infected].InitialValue"}\right)$$

The resulting data were captured manually in the excelfile ‘Fig 3. Government measure versus resultsNEW’, copied to ‘Figure 3 Excel’.

Files are on the GitHub https://github.com/HansWesterhoff/Coronapaper-March-2020

The figure 3 is:

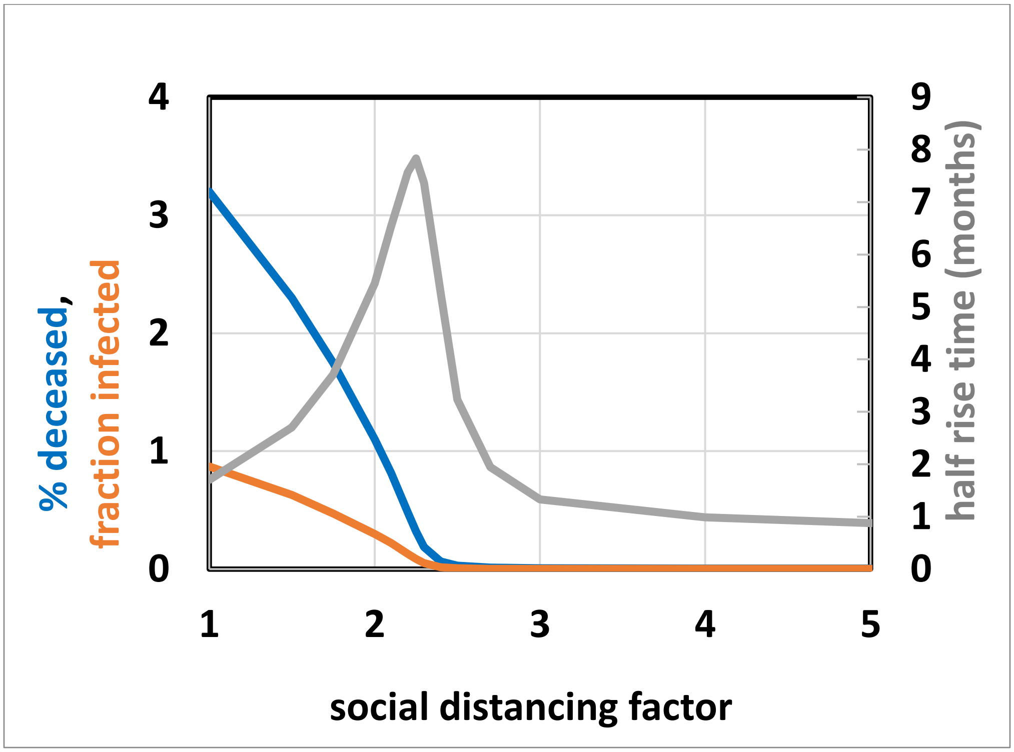

Figure 4 Intermittent lockdown

Filename: Wff1Corona_Lux_2020-03-20_1340 intermittentGwork full lockdown effect of socialdistancing Fig 3 also Fig 2WORK2A.cps renamed to Figure 4 Copasi.cps.

Settings: Time fraction lockdown = 0.7 for Fig 4B and 0.55 for Figure 4A, Lockdown duration = 7, Government_induced_isolation_factor = premultiplier 10:

$$10\cdot\left(1+\frac{\frac{2.5\cdot 141}{141}\cdot\text{"[symptoms-tested]"}}{\text{"Values[symptomatic number tolerance].InitialValue"}}\cdot\text{"Values[government response factor to diagnosed infected].InitialValue"}\right)$$

Excel file: Fig4ANewWff1Corona_Lux_2020-03-20_1340 intermittentGwork full lockdown effect of socialdistancing Fig 4 intermittentWORK and Fig4BNewWff1Corona_Lux_2020-03-20_1340 intermittentGwork full lockdown effect of socialdistancing Fig 4 intermittentWORK

Renamed to Figure 4A Excel and Figure 4B Excel.

Filename: Wff1Corona_Lux_2020-03-20_1340 intermittentGwork full lockdown effect of socialdistancing Fig 3 also Fig 2WORK2A.cps, renamed to Figure 4 Copasi.

Settings:

Time fraction lockdown = 0.7 for Fig 4B and 0.55 for Figure 4A

Lockdown duration = 7

Files are on the GitHub https://github.com/HansWesterhoff/Coronapaper-March-2020

The figure 4 is:

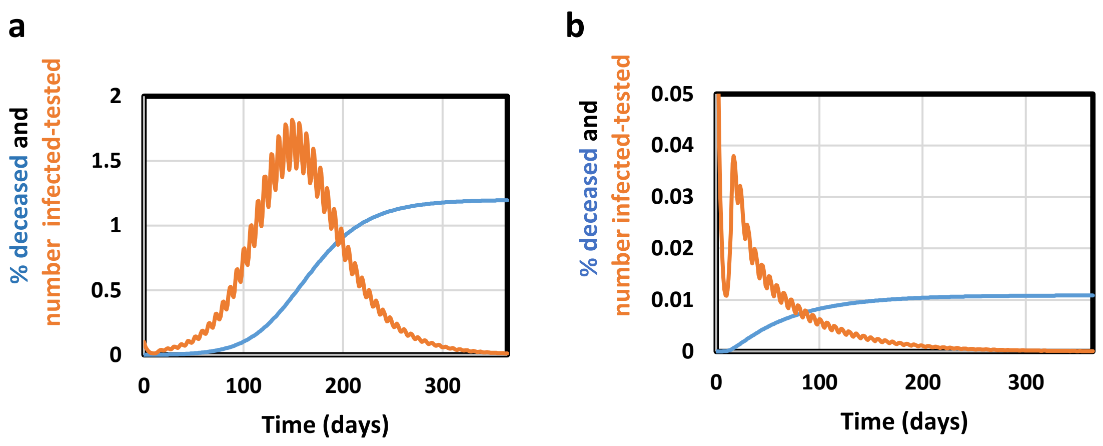

Figure 5. Adaptive lockdown

Full lockdown setting except that the social distancing factor is modulated as a function of symptoms-tested.

Figure 5 files. Full lockdown setting except that the social distancing factor is modulated as a function of symptoms-tested

Excel file: Fig NEW 5 inf tested, govern, % dead, sympt testedtext copied to 'Figure 5 Excel'

Copasi file: Wff1Corona_Lux_2020-03-20_1340 intermittentGwork full lockdown effect of socialdistancing Fig 5 adaptive copied to 'Figure 5 Copasi'. Files are on the GitHub https://github.com/HansWesterhoff/Coronapaper-March-2020

The figure 5 is:

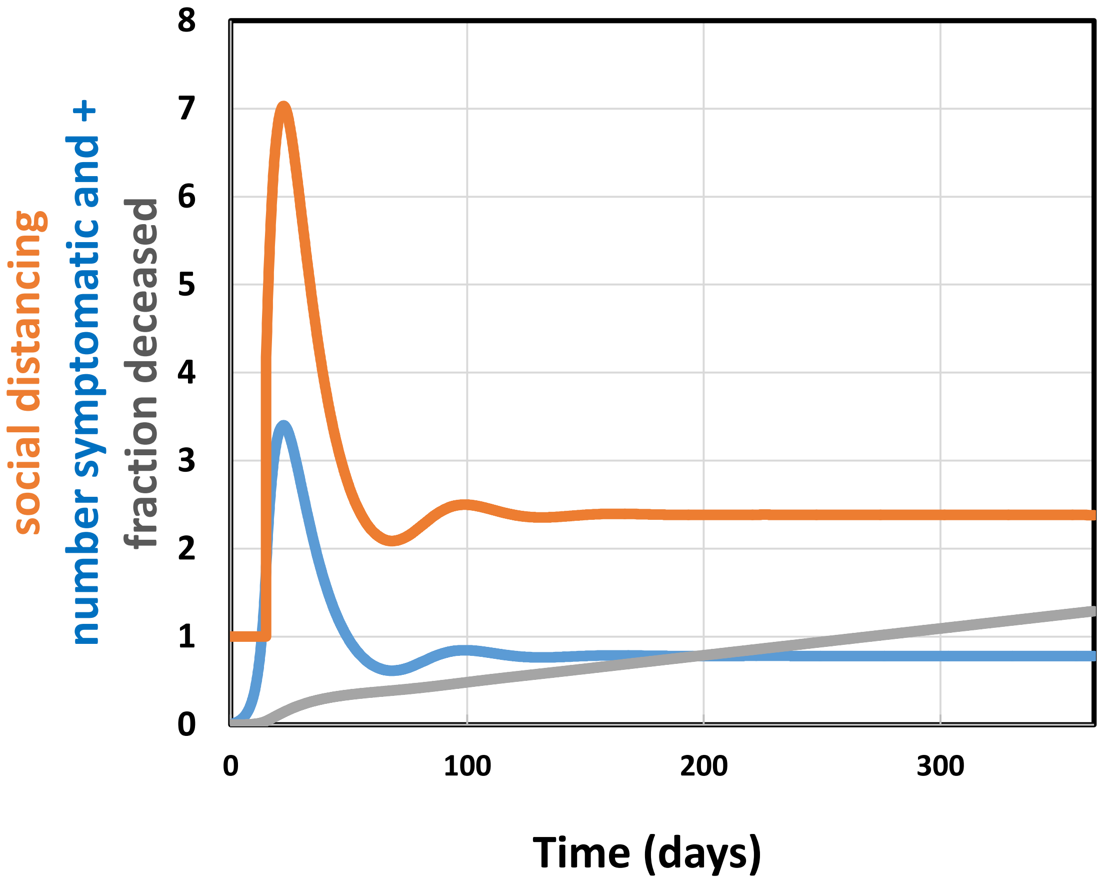

Figure 6. Strong then soft better than soft then strong

Copasi filename: Previously called 'Fig S4 attempts at strong then soft and vice versa.' Now copied to 'Figure 6 Copasi.cps'. Global quantities 'first social distancing factor factor' and 'second social distancing factor factor' were set to 10 and 2, or 2 and 10 respectively and then a time integration was run, data saved into excel files and combined. Excel file: Figure 6 Excel. Files are on the GitHub https://github.com/HansWesterhoff/Coronapaper-March-2020

The figure 6 is:

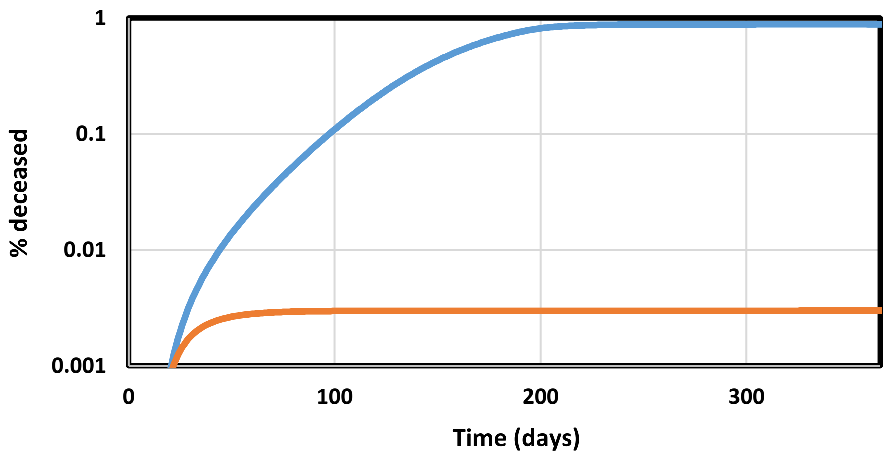
1
0.1
0.01
0.001
% deceased
0
100
200
300
Time (days)
